# Effects of intensive and conventional farming on oxidative stress and meat quality biomarkers in holstein and simmental cattle

**DOI:** 10.1038/s41598-024-78087-x

**Published:** 2024-10-31

**Authors:** Ali Murat Tatar

**Affiliations:** Faculty of Agriculture, Department of Animal Science, Dicle University, 21280 Diyarbakır, Turkey

**Keywords:** Oxidative stress, Meat quality, Antioxidant gene response, Intensive farming, Apoptotic proteins, Molecular biology, Zoology, Animal behaviour

## Abstract

This study investigates the intricate factors influencing meat quality, including breed, rearing conditions, and processing, with a primary focus on oxidative stress in Holstein Friesian and Simmental cattle within conventional and intensive production systems. A notable difference in oxidative stress was found between animals subjected to intensive-farming versus conventional practices, with Holstein cattle showing a more pronounced antioxidant gene response than Simmental. The analysis revealed that intensive rearing conditions resulted in increased DNA repair activity and expression of stress-response proteins like heat shock proteins, suggestive of greater cellular damage and an adaptive stress response. Muscle tissue analyses, revealed a clear distinction in gene expression associated with meat quality between the breeds and the type of farming system. A negative correlation emerged between oxidative stress levels and genes related to muscle development, which affects meat quality. Intensive farming conditions altered the expressions of apoptotic proteins, impacting meat quality at the molecular level. These results underscore the profound effect rearing conditions have on meat quality, driven by stress-related molecular responses. This highlights the need for further research into the influence of husbandry practices on animal welfare and meat quality, with the intention of developing strategies to mitigate the negative consequences of intensive-farming.

## Introduction

Within the paradigm of sustainable animal husbandry, the utilization of specialized breeds within intensive meat production regimes holds promise for diminishing resource inputs, notably feed, curtailing nutrient losses, and mitigating deleterious emissions such as methane and nitrogenous compounds, thereby enhancing the efficiency of meat output relative to traditional farming practices and indigenous breeds. On the contrary, employing low-quality forage may find greater suitability within populations of landraces or crossbreeds, as well as within the frameworks of traditional breeding methodologies. Accordingly, divergent agricultural systems can engender a spectrum of trade-offs, spanning the conservation of biodiversity and facilitation of carbon sequestration to the optimization of feed conversion ratios^1–3^. In light of these considerations, the scientific community recognizes an imperative for amplified research and empirical comparative analyses to elucidate the relative merits of conventional versus intensive breeding strategies concerning sustainable livestock management and meat quality^4,5^.

Conventional farming, often associated with traditional or extensive agriculture, significantly differs from modern intensive practices. Extensive farming typically involves the use of natural resources such as forage and water, with livestock grazing freely. On the other hand, intensive agriculture represents a modern approach, rapidly expanding to provide almost 40% of the world’s meat. In this type of farming, animals are densely housed and fed high-energy, grain-based diets with synthetic chemicals and additives to promote growth. Advanced technologies like computer software, animal tracking systems, robotic feed distribution, and watering systems are being regularly utilized. While these technologies offer opportunities for animals, they also bring health issues associated with the modern age to the forefront^6–8^. Consumers’ growing health awareness has raised concerns. There is a preference for leaner, less fatty meats, highlighting the conflict between the delicious taste of fats and the health risks they pose, such as heart disease. Meat from intensive systems is high in omega-6 fatty acids, which can increase this risk, while meat from extensive systems is richer in beneficial omega-3 s^9^. Another key driver for consumers is the demand for sustainable and ethical farming practices. Animal welfare, once a contentious issue, is now a top priority, with many consumers willing to pay more for meat with humane certification, though this may not always translate to purchasing behaviour. Ultimately, both intensive and extensive farming practices have their advantages and disadvantages. Factors such as meat quality, animal welfare, and environmental impact are increasingly influencing consumer preferences, challenging the meat production industry to adapt and find a balance between these considerations^10,11^. In this regard, the recognition of the effects that approximately two years of rearing conditions have on meat quality at the final destination, slaughterhouses, is becoming increasingly critical. Furthermore, the understanding of the factors that contribute to these effects is gaining importance with each passing day.

Optimization of meat quality in bovine production necessitates a comprehensive evaluation of the entirety of the production continuum, ranging from breeding methodologies to meat processing techniques. Ostensibly, there exists an intrinsic association between farm management practices, the health and morbidity profiles of livestock, and the resultant quality of meat products. Empirical evidence from an array of systematic investigations corroborates the assertion that meat quality serves as a pivotal element within the framework of sustainable livestock farming paradigms, which prioritize product excellence over sheer volume of output^12^. This model manifests a judicious approach in comparison to intensive production systems, which are oftentimes correlated with an uptick in disease incidence, notably cancer, as well as food security concerns. Furthermore, sustainable and natural practices have been acknowledged for their alignment with the preferences of discerning consumers whose dietary choices are swiftly evolving^13,14^.

Exploratory research within this domain has addressed the significance of numerous determinants on meat quality potential, encompassing breed genetics^15,16^, rearing conditions^17–19^, and animal nutrition^20,21^. Additionally, attributes at the moment of slaughter^22,23^, antemortem stressors^24^, and postmortem meat processing parameters^14,25^ have been scrutinized for their impacts.

Among these factors, oxidative stress has been identified as a considerable influence on meat quality, intersecting with metabolic, physiological, and genetic mechanisms within the meat matrix. The presence of oxidizing agents, high levels of polyunsaturated lipids, heme moieties, and metallic catalysts in muscular tissue predisposes meat to oxidative deterioration. This degradation process is evidenced through alterations in meat coloration, flavor degradation, production of harmful compounds, reduction in shelf stability, and diminution of nutritive value^26,27^.

The phenomenon of oxidative stress delineates a detrimental state whereby oxidative damage precipitates an imbalance skewed toward the generation of free radicals over the capacity of antioxidant defenses^28–30^. This state of redox disequilibrium can be induced by various exogenous stressors that disrupt an animal’s homeostasis prior to slaughter^31^. Such stressors predominantly arise during transport, where factors including suboptimal handling practices, inadequate road infrastructure, rapid or irregular vehicular motion, extensive transport distances, nutritional deprivation, interspecies confinement, and instances of aggressive handling are implicated in the genesis of oxidative stress^32–35^. Environmental elements such as divergent air flow, extreme temperature, variable humidity, and acoustic disturbances further contribute to the animal’s stress load^33,36,37^.

Consequential to transport-related upheavals, there is an adverse impact on animal welfare, which manifests as physical discomfort, alterations in typical behaviors, and subsequent negative implications for meat quality^38^. At the physiological level, oxidative stress may evoke anomalies in cardiac rhythm, arterial pressure, and thermoregulation. This stress response activates haphazard secretion of cortisol and catecholamines, culminating in glycogen exhaustion and the manifestation of dark, firm, and dry (DFD) meat conditions^39,40^. The process is further characterized by an overproduction of reactive oxygen species (ROS) within the musculature and an influx of stress hormones into the bloodstream^41,42^. Subsequent lipid peroxidation within muscle tissue, a byproduct of oxidative stress, undermines meat quality via adverse effects on the nutritional and organoleptic attributes of the meat products^43–45^.

Recent advancements in genomics and bioinformatics have facilitated a more nuanced exploration of biological processes that are pivotal to meat quality. These processes encompass gene expression profiles, physiological adaptations, enzymatic activities, and broader metabolic pathways^16,24,25^. In such investigations, genes that act as biomarkers for oxidative stress, DNA integrity perturbations, molecular chaperone systems, inflammatory responses, and apoptotic pathways have been delineated, predominantly in humans and model organisms^46,47^. Concurrently, molecular biomarkers indicative of meat quality traits shaped by genetic lineage, muscular attributes, husbandry practices, and post-mortem processing conditions have commenced to be characterized, elucidating their implications on animal growth and metabolism^48,49^.

In this investigation, we scrutinized the effects of conventional and intensive rearing systems on oxidative stress-dependent molecular signaling pathways during and post-mortem in two extensively cultivated, high-yielding bovine breeds—Holstein and Simmental. The hypothesis postulates that the stresses encountered during pre-slaughter phases, especially those associated with transportation to and holding at the abattoir, significantly impact the quality of meat post-mortem. The primary objective is to delineate the extent to which pre-slaughter stress influences the molecular determinants of meat quality. Through this lens, the purpose of the study is to delineate the potential implications of these stressors on post-mortem meat quality and to utilize the outcomes as a means to discern the potential benefits or detriments that conventional versus intensive farming practices may impact on the quality of meat products.

## Results

### Oxidative stress and antioxidant defence signals in blood tissue

The malondialdehyde (MDA) levels, and fluorescence signals produced by the CM-H_2_DCFDA revealed that across all groups, there was a significant elevation of MDA levels and green fluorescence intensity in comparison to the control group, indicating heightened oxidative stress.

**Fig. 1:**
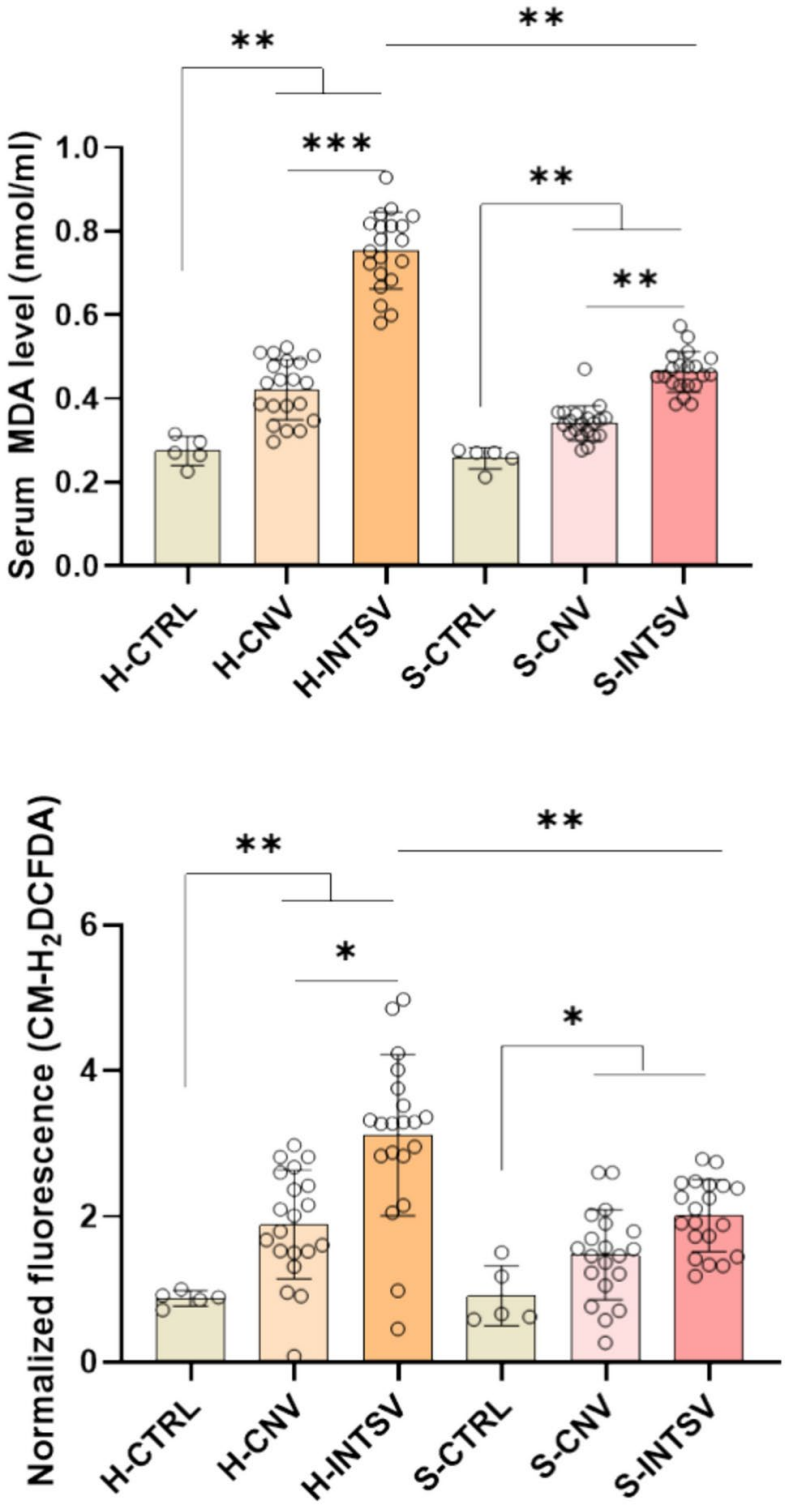
Comparative Analysis of Malondialdehyde (MDA) and CM-H2DCFDA Levels in Post-Slaughter Serum samples of Holstein (H) and Simmental (S) Bovines Reared under Conventional and Intensive Production Systems. Data are presented as Mean ± SD (n = 20). Asterisks denote statistically significant differences between means as determined by one-way ANOVA followed by Tukey’s HSD test, with *P ≤ 0.05, **P ≤ 0.01, and ***P ≤ 0.001. CTRL: Control, CNV: Conventional, INTSV: Intensive.

The highest levels of oxidative stress were documented in animals from farms practicing intensive agriculture. In contrast, animals raised under conventional farming exhibited significantly lower levels of oxidative stress compared to their intensively farmed counterparts. Furthermore, when comparing the two breeds, Simmental cattle showed a reduced profile of oxidative stress, characterized by lower MDA levels and a diminished green fluorescence signal elicited by CM-H_2_DCFDA (Fig. 1).

**Fig. 2:**
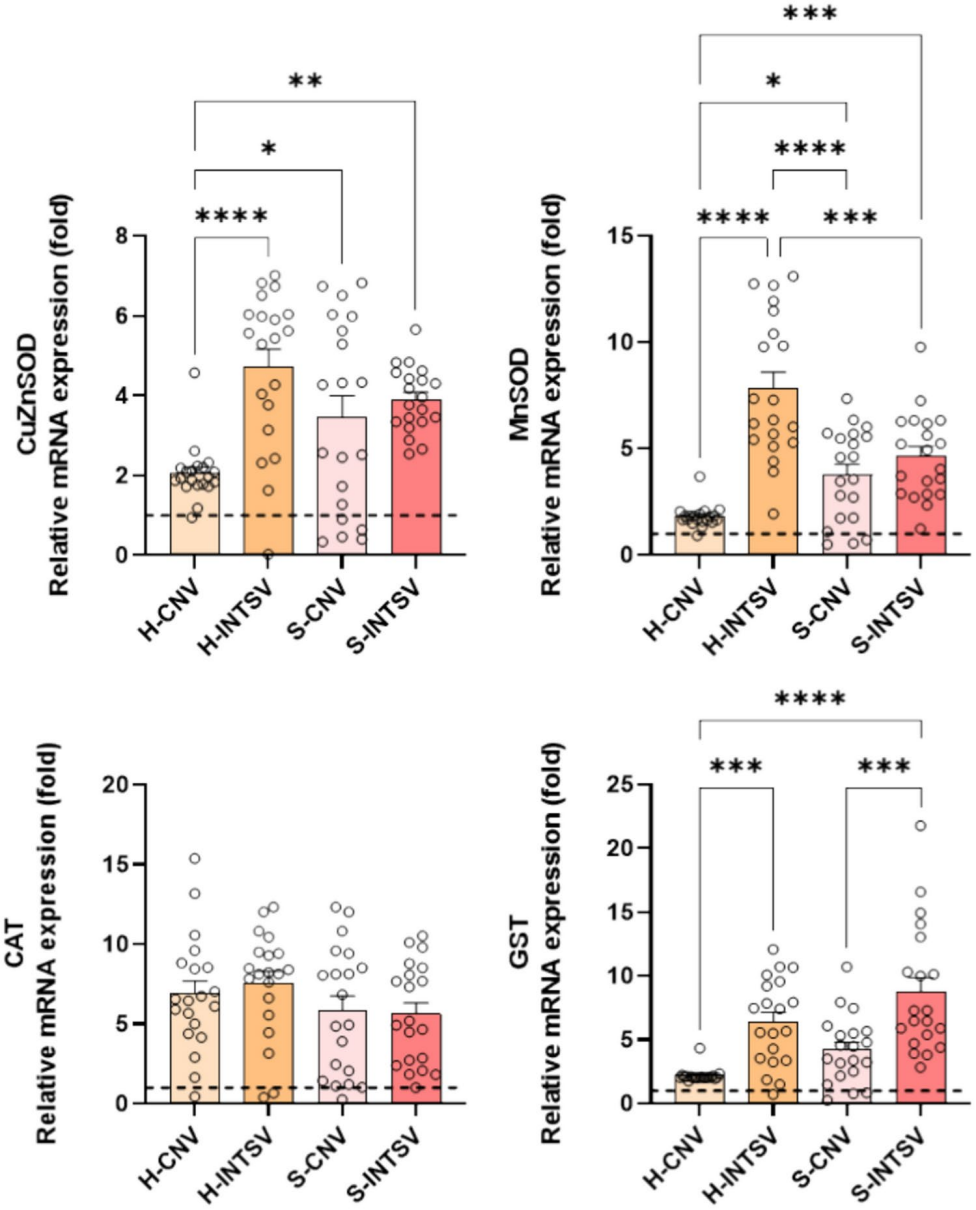
Relative fold change determined by quantitative real-time PCR (qRT-PCR) analysis of antioxidant defence genes in Post-Slaughter Serum samples of Holstein (H) and Simmental (S) Bovines Reared under Conventional and Intensive Production Systems. All data were normalized with multiple control using GAPDH and β-actin expression and are given as relative to control (control = 1 and is shown as a dashed line). Data are presented as mean ± SE, n = 20. Asterisks denote statistically significant differences between means as determined by one-way ANOVA followed by Tukey’s HSD test, with *P ≤ 0.05, **P ≤ 0.01, and ***P ≤ 0.001. CTRL: Control, CNV: Conventional, INTSV: Intensive.

Simultaneously, oxidative stress was tracked by examining the expression of antioxidant genes in the blood tissues of the same animals. The expression levels of CuZn-SOD, MnSOD, and GST were found to be notably elevated in Holstein cattle from intensive farming practices compared to other groups. No significant difference in antioxidant gene expression was observed between intensive and conventional farming in the Simmental breed. However, expressions of all antioxidant genes were higher in both groups when compared to the control (Fig. 2).

### DNA repair and heat shock proteins responses

**Fig. 3:**
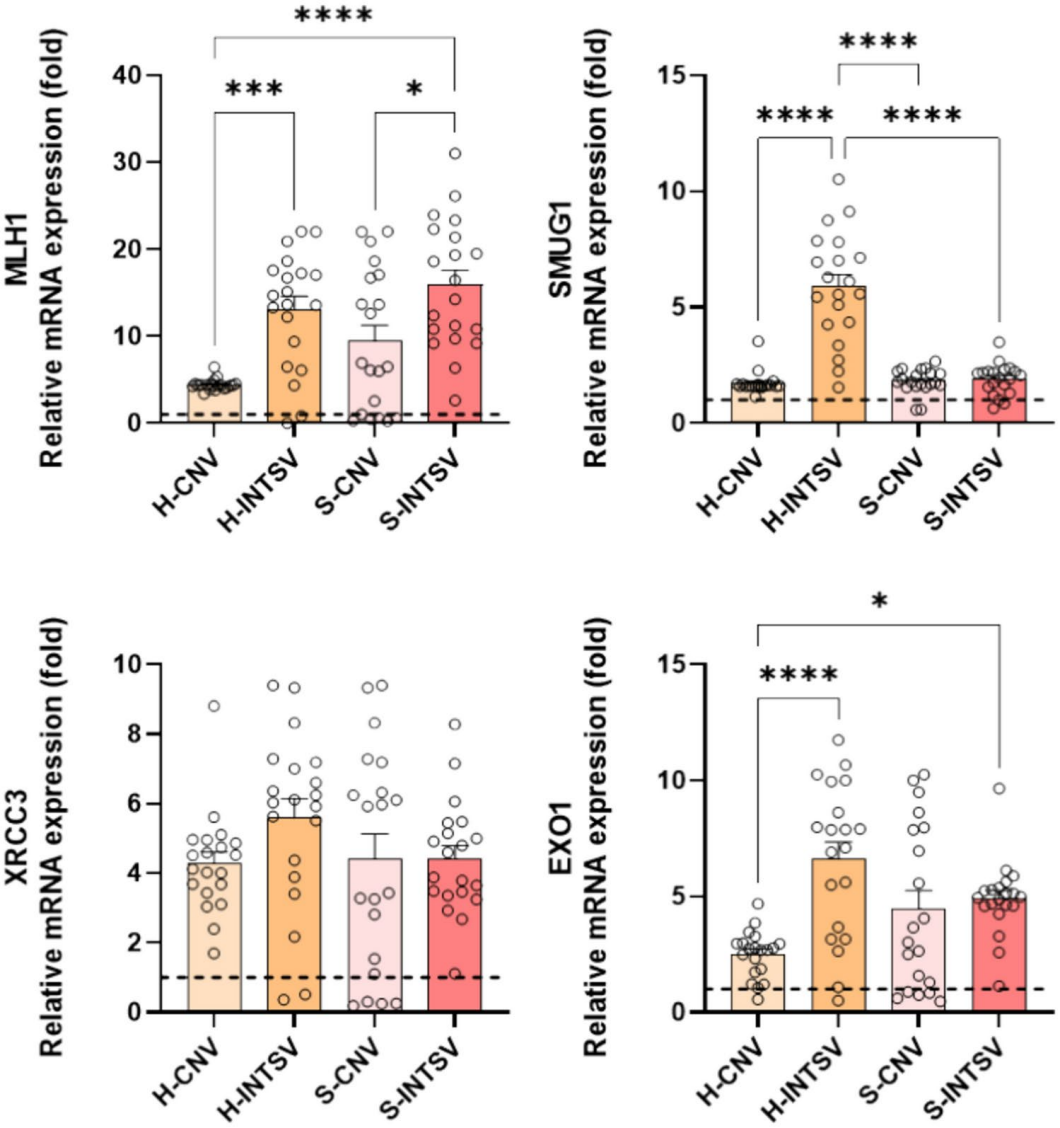
Relative fold change determined by quantitative real-time PCR (qRT-PCR) analysis of DNA repair pathway genes in Post-Slaughter Serum samples of Holstein (H) and Simmental (S) Bovines Reared under Conventional and Intensive Production Systems. All data were normalized with multiple control using GAPDH and β-actin expression and are given as relative to control (control = 1 and is shown as a dashed line). Data are presented as mean ± SE, n = 20. Asterisks denote statistically significant differences between means as determined by one-way ANOVA followed by Tukey’s HSD test, with *P ≤ 0.05, **P ≤ 0.01, and ***P ≤ 0.001. CTRL: Control, CNV: Conventional, INTSV: Intensive.

The study also probed the effects of sustained stress on cellular components. An excessive accumulation of reactive oxygen species (ROS), beyond the neutralizing capacity of antioxidant defenses, can attack critical cellular molecules, such as DNA, proteins, membrane lipids, and vesicles. Potential DNA damage in both breeds was assessed through the expression of DNA repair genes, along with protein damage responses associated with misfolded or unfolding proteins, as indicated by the expression profiles of the Heat Shock Protein family (HSPs). The findings indicated an increase in DNA repair activity across both breeds when contrasted with the controls. Notably, gene expression analysis revealed a significant increase in the expressions of MLH1, SMUG1 and EXO1 in Holstein cattle from intensive farming, while expressions of MLH1 were significant in the Simmentals (Fig. 3).

**Fig. 4:**
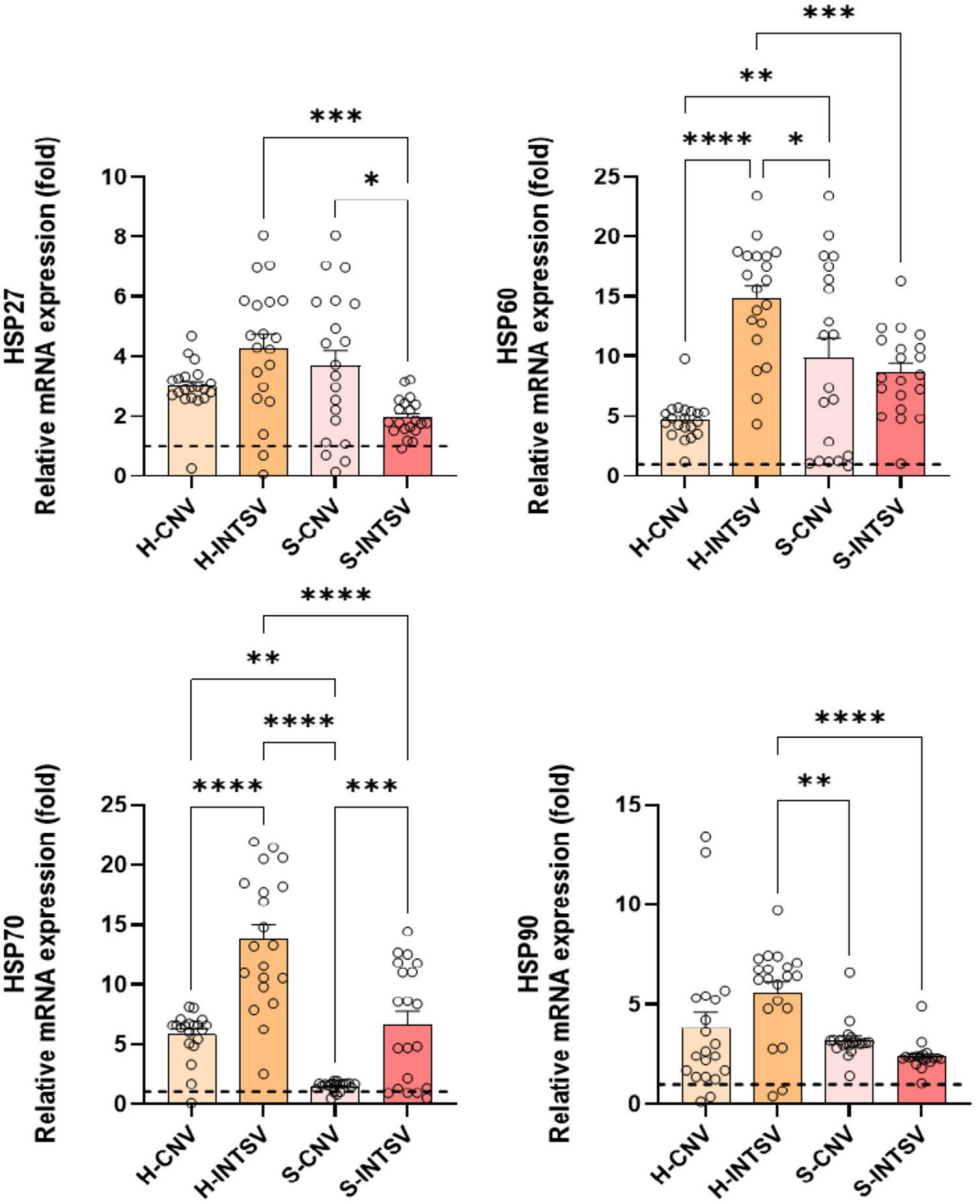
Relative fold change determined by quantitative real-time PCR (qRT-PCR) analysis of HSPs family genes in Post-Slaughter Serum samples of Holstein (H) and Simmental (S) Bovines Reared under Conventional and Intensive Production Systems. All data were normalized with multiple control using GAPDH and β-actin expression and are given as relative to control (control = 1 and is shown as a dashed line). Data are presented as mean ± SE, n = 20. Asterisks denote statistically significant differences between means as determined by one-way ANOVA followed by Tukey’s HSD test, with *P ≤ 0.05, **P ≤ 0.01, and ***P ≤ 0.001. CTRL: Control, CNV: Conventional, INTSV: Intensive.

Additionally, the overexpression of stress-specific HSP70, indicative of stress response, was characteristic of both groups under both farming conditions. Alongside this, HSP90 and mitochondrial HSP60, biomarkers of mis/un-folded protein and mitochondrial damage, respectively, were significantly higher in Holstein population subjected to intensive farming practices as compared to those from conventional farming setups (Fig. 4).

### Meat quality-related gene expression profile in post mortem Longissimus dorsi muscle tissues

**Fig. 5:**
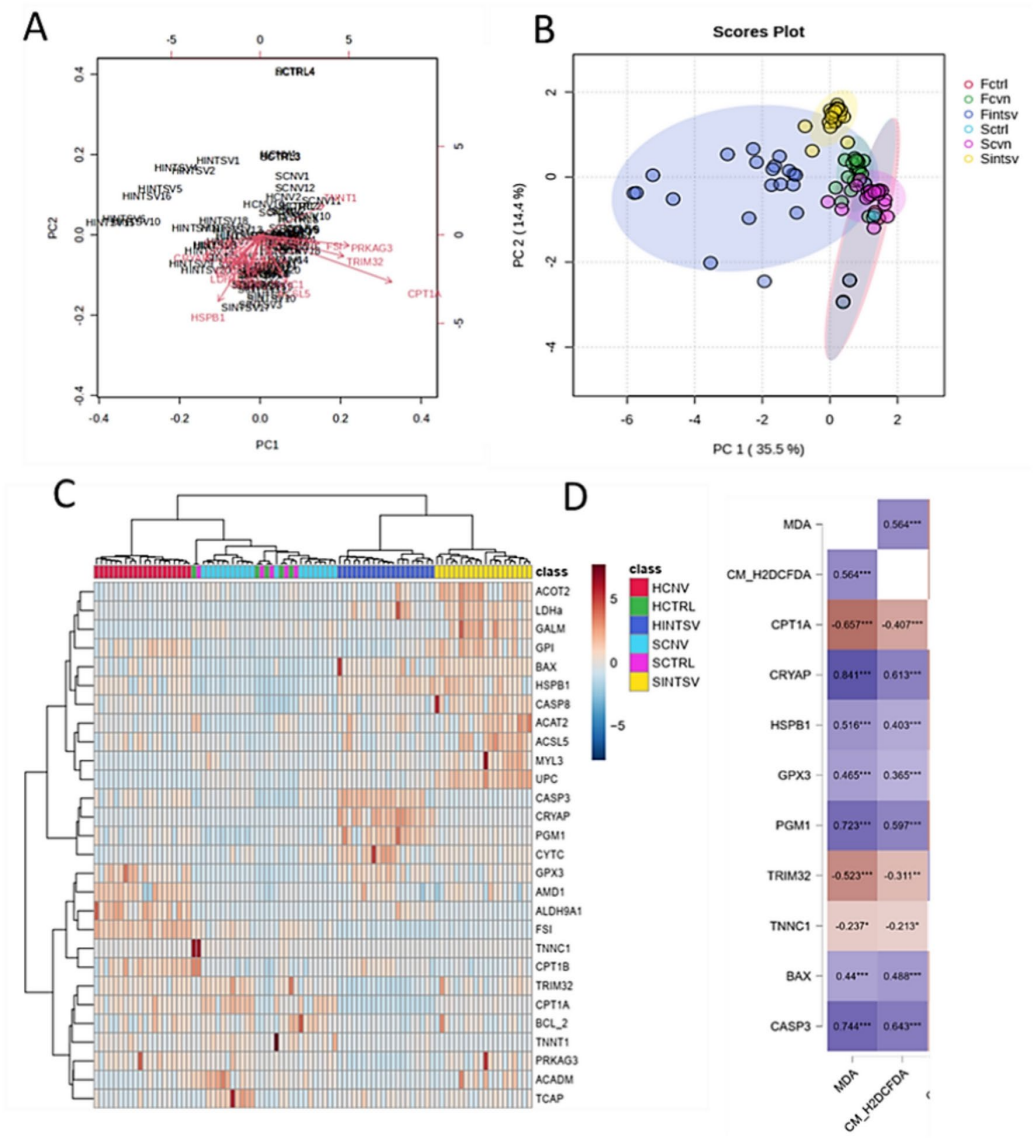
(**A**) Vectors of meat quality genes distributed on the coordinate system affected by the first two factors resulting from principal component analysis (PCA). (**B**) Principal component analysis (PCA) of the expressed genes indicating predominant molecular signals associated with the fatty acid composition, meat tenderness, and shear force characteristics of the Longissimus dorsi muscle. The analysis reveals distinct expression profiles and levels, differentiating between Holstein (H) and Simmental (S) populations reared under conventional (CNV) and intensive (INTSV) production systems. CTRL denotes the control group. (**C**) Heat map analysis using gene expression levels of Longissimus dorsi muscle of Holstein and Simmental groups. Euclidian correlation options and Ward’s linkage (clustering to minimize the sum of squares of any two clusters) were used to agglomerate hierarchical clustering. (**D**) Pearson correlation coefficients and significance levels for the expression of meat quality-related genes and their significant associations with serum oxidative stress parameters, including malondialdehyde (MDA) and CM-H2DCFDA.

In the subsequent phase of the investigation, the focus shifted to the impact of systemic oxidative stress, as determined in blood tissue, on the expression of genes previously proposed as biomarkers of meat quality in comprehensive transcriptomic studies. To achieve this objective, the evaluation encompassed the expression of 28 genes implicated in pathways related to muscle development, oxidative stress, glucose metabolism, lipid mechanisms, shear force, and apoptosis in post-mortem Longissimus dorsi muscle tissues from both Holstein and Simmental populations reared under control, conventional, and intensive farming conditions. Through principal component analysis (PCA) utilizing the mentioned gene expression data, a clear segregation between the Holstein and Simmental populations bred in intensive production systems was discerned, with each clustering within distinct regions of the biplot (Fig. 5a, b). Concurrently, heat map analysis confirmed this separation, presenting two distinct hierarchical clusters representing intensive and conventional production groups for both cattle populations. Interestingly, the conventional production groups were closely associated with the control group in this analysis (Fig. 5c). Critical differentiation within this partitioning was largely attributed to genes such as HSPB1, CRYAB, CPT1A, PRKAG3, TRIM32, TNNT1, as well as several genes within the apoptosis pathway. Further correlative assessments via Pearson correlation analysis were employed to explore the relationship between serum levels of oxidative stress markers, MDA and CM-H2DCFDA, and the expression of meat quality genes. The results suggested a negative correlation between serum oxidative stress markers and key indicators of muscle development (CPT1A), meat tenderness (TRIM32 and PRKAG3), as well as shear force (TNNT1). Notably, a significant positive correlation was observed particularly among genes serving as biomarkers for oxidative stress and apoptosis (Fig. 5d).

**Fig. 6:**
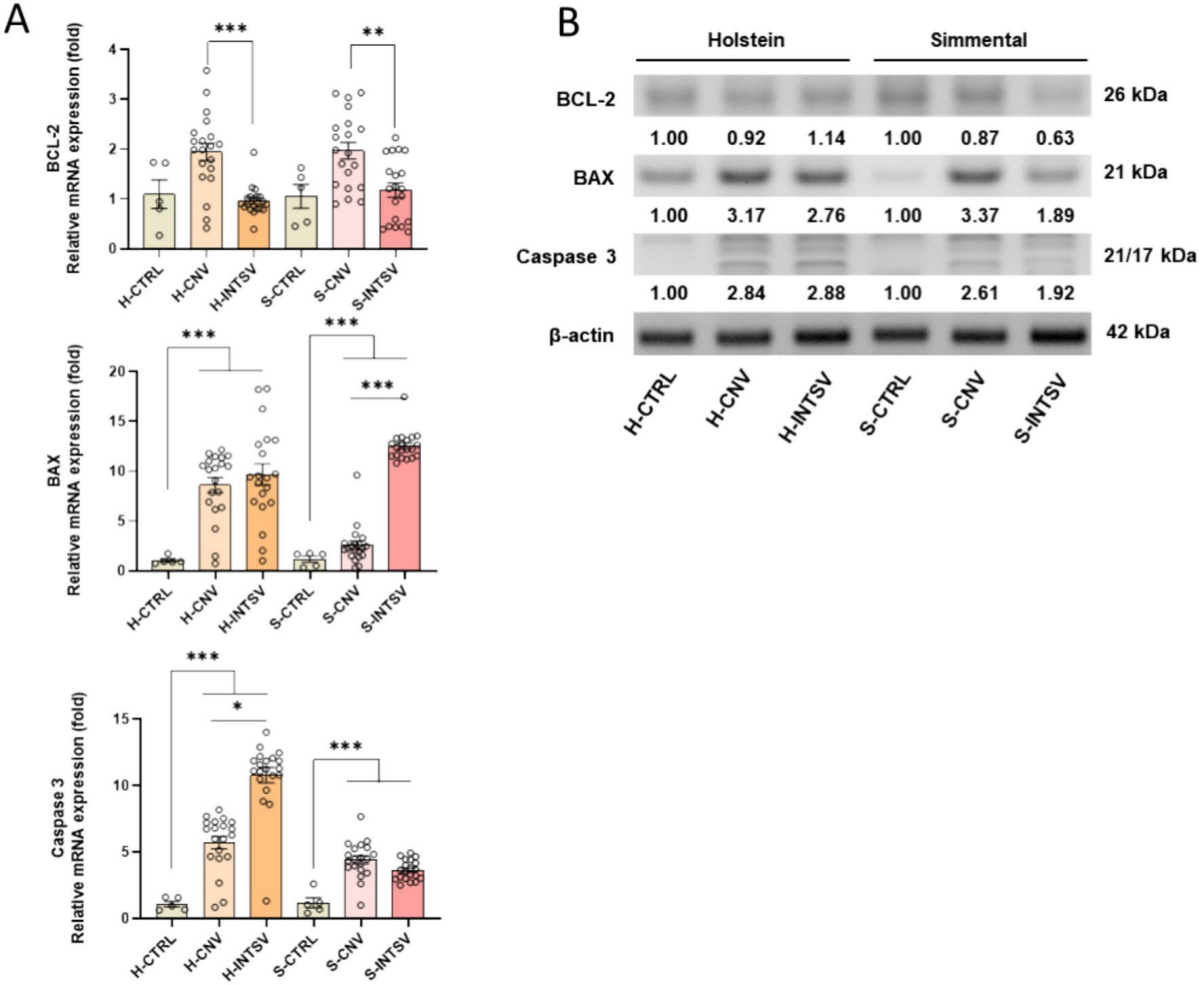
(**A**) Relative fold change determined by quantitative real-time PCR (qRT-PCR) analysis of BCL-2, BAX AND Caspase 3 genes belong to Mitochondrial apoptosis pathway in Post-Slaughter Serum samples of Holstein (H) and Simmental (S) Bovines Reared under Conventional and Intensive Production Systems. All data were normalized with multiple control using GAPDH and β-actin expression and are given as relative to control. Data are presented as mean ± SE, n = 20. Asterisks denote statistically significant differences between means as determined by one-way ANOVA followed by Tukey’s HSD test, with *P ≤ 0.05, **P ≤ 0.01, and ***P ≤ 0.001. (**B**) Western blot analysis of BCL-2, BAX and cleaved caspase-3 (relative density normalized with β-actin). CTRL: Control, CNV: Conventional, INTSV: Intensive.

Consequently, to delve further into this phenomenon, the levels of proteins identified as apoptotic biomarkers BCL-2, BAX, and Caspase-3 – were assessed from the total protein extracts pooled from Longissimus dorsi muscle tissues of the bovine subjects. This was undertaken for both Simmental and Holstein breeds reared under conventional and intensive farming conditions. Comparison of these protein levels with the gene expression parameters revealed discernible trends. Quantitative real-time PCR (qPCR) analysis indicated that, compared to conventional rearing, the intensive production setting resulted in diminished levels of the anti-apoptotic protein BCL-2 in both breeds. Conversely, expression of the pro-apoptotic proteins BAX and Caspase-3 was significantly elevated compared to the control group, with the highest expression of BAX being notable in the Simmental population under intensive production, and the most pronounced expression of Caspase-3 detected in the Holstein population subjected to the same intensive production conditions (Fig. 6A). Western blot analysis corroborated the gene expression data, confirming the induction of significant apoptotic signalling within the muscle tissues of both breeds (Fig. 6B).

## Discussion

The study suggests the crucial emphasis on the importance of high-quality, nutrient-dense meat with an extended shelf life as a critical element of human food security. The continuous growth in the global population, coupled with the effects of climate change, poses ongoing challenges to meat production, making the attainment of high-quality meat more complex. This challenge is further compounded by the significant increase in diseases such as cancer, obesity, diabetes, and undiagnosed autoimmune disorders. A notable shift in consumer demand toward healthy and natural foods, particularly meats and meat products, is now becoming a decisive factor for production systems. Consequently, a primary concern is ensuring the maintenance of meat quality and shelf life during processing in slaughterhouses. These facilities represent the final stage before meat reaches the consumer, where any adverse effects on meat quality become irreparable.

Existing research has demonstrated that post-slaughter meat performance is influenced by factors such as animal breed, variations in rearing conditions, transport conditions to the slaughterhouse, conditions in holding areas before slaughter, and the methods of slaughter. Notably, transporting animals to slaughterhouses can introduce significant thermal stress, and confining animals in tight, enclosed spaces, along with exposure to noise, vibration, and wind, can result in substantial stress and physiological alterations^32,33,35^. Furthermore, conditions at holding facilities, including encounters with unfamiliar animals, immobilization in confined spaces, and changes in access to food and water, elicit a strong stress response. The effect of these conditions on meat quality has been thoroughly investigated and remains a subject of intense scientific inquiry^33,36^. All the years of labor, numerous scientific studies, and specialized production systems are dedicated to producing the highest quality, healthiest, and longest-lasting meat products up until the critical moment of slaughter.

The studies indicated that the stresses experienced, particularly during transport and in holding areas, triggered a significant accumulation of reactive oxygen species (ROS) and an increase in the lipid peroxidation response in cattle^38–40^. Consistent with these findings, both the MDA analysis and CM-H2DCFDA staining results of post-mortem serum samples pointed to a significant increase in lipid peroxidation and oxidative stress across both rearing conditions and cattle breeds when compared to control samples.

To gain a deeper understanding of how the oxidative stress identified in serum samples translated to the tissues, findings were expanded with molecular data. Increased ROS and resultant oxidative stress initially activate the antioxidant system within cells and tissues. The antioxidant defense system, utilizing mechanisms such as superoxide dismutases, peroxidases, catalase, and glutathione pathways, seeks to scavenge or neutralize these ROS in various ways. qRT-PCR analysis findings indicated that antioxidant gene expression significantly increased in both breeds and rearing conditions compared to controls. Specifically, the expression levels of cytosolic enzyme CuZn-SOD and mitochondrial antioxidant Mn-SOD were significantly lower in Holstein cattle reared on pasture compared to those under intensive conditions. Regarding GST gene expression, this difference was found to be significant in both cattle breeds.

An excessive accumulation of ROS, beyond the neutralizing capacity of antioxidant defenses, can attack critical cellular molecules, such as DNA, proteins, membrane lipids, and vesicles. DNA repair genes serve as rapid and effective biomarkers of DNA damage^50–53^. In line with previous studies, findings particularly point to increased post-mortem blood tissue levels of MLH1 (base excision repair), SMUG1 (single and double-strand repair), and EXO1 (double-strand repair) genes in Holstein cattle, indicative of base excision and single and double-strand DNA damages. In Simmental cattle, the damage was particularly noted in the context of base excision.

If the DNA damage that occurs within the cellular environment is not repaired, proteins synthesized during translation could misfold or fail to fold at all. Such proteins may not only be dysfunctional but can also be detrimental to the cell. Misfolded or unfolded proteins in the cell are typically refolded by molecular chaperones known as Heat Shock proteins or are degraded if they are irreparable. Thus, the increase in DNA damage-related markers is also considered a biomarker for protein damage^46,47,54^. HSP70 is a stress-specific molecular chaperone^55^. Study results revealed a significantly higher expression of HSP70 in both breeds and rearing conditions compared to controls. Additionally, cattle raised in intensive production systems exhibited significantly higher chaperone activity compared to those in conventional systems. Alongside this, both mitochondrial HSP (HSP60) and the unfolded protein response biomarker HSP90 levels were higher in Holstein compared to Simmental cattle, and significantly higher gene expressions were determined in intensive production compared to conventional. Taken as a whole, blood tissue findings indicate that, in both Holstein and Simmental cattle, the conditions of transport and holding areas prior to slaughter led to significantly higher levels of molecular damage associated with oxidative stress compared to controls. This divergence was notably more pronounced in Holstein cattle and also more marked in both breeds under intensive production systems.

Blood tissue responds to stress with its cells, yet it also participates in the physiological processes of gene expression occurring throughout the body. These processes culminate at their site of origin, whereafter the completed mRNAs, once their expression-triggering conditions no longer exist, are expelled from the cell and enter the bloodstream. Consequently, gene expressions identified in total blood tissue provide significant insight into physiological processes occurring body-wide, as delineated by Vardar et al. in 2021^56^. In the study, the molecular signal related to oxidative stress identified in blood tissue is believed not to be confined to just blood tissues but reflects overall physiological conditions throughout the body.

In mammals, high and sustained oxidative stress, the consequent lipid peroxidation, irreversible DNA damage, and the responses of unfolded/misfolded proteins induce programmed cell death to prevent this damage from reaching new generations, culminating in apoptosis. Post-mortem or even preceding it, apoptosis in meat tissues is directly related to meat quality and has detrimental effects on it^51,54,57^. In keeping with these findings, the study observed that while the expression of the apoptotic inhibitor BCL-2 remained unchanged in all groups where oxidative stress was notably high compared to controls, there was a significant increase in the expression of the pro-apoptotic gene BAX, responsible for mitochondrial membrane potential disruption, and the expression of Caspase 3, an effector protein of apoptosis. The Western blot analysis supported the gene expression data at the protein level, indicating a move towards a strong apoptotic signal. Overall, a higher pro-apoptotic expression was detected in the Holstein breed and particularly under intensive production systems. This data suggests that within meat from cattle, apoptosis occurs and is associated with pathophysiological processes induced by oxidative stress.

In the concluding section of the study, focus was placed on the molecular effects of oxidative stress and the ensuing pathologies identified in the experimental groups on gene expressions related to meat quality. The relationship between oxidative stress and meat quality has been explored in only a handful of studies. It has been observed that myofibrillar proteins are impacted by ROS during the maturation and preservation of meat. Additionally, high levels of free radicals and ROS have been reported to lead to degenerative damage to cellular structures and subsequently affect meat quality^42^. The influence of oxidative stress on meat tenderness has also been substantiated. Evidence has been presented that demonstrates the effect of ROS on the reduction of intramuscular collagen due to the balance between the degradation by matrix metalloproteinase-2 (MMP-2) enzyme and the synthesis by intramuscular fibroblasts from bovine muscles^58^ The findings indicate that ROS increases MMP-2 activity and decreases collagen synthesis. This reduction in collagen synthesis diminishes collagen solubility, which, in turn, results in increased toughness of the meat^59^.

Recent years have seen an intensified research focus on transcriptomic molecular biomarkers that could potentially reveal meat quality attributes. Strong evidence has emerged suggesting that gene expressions associated with the physiological processes of meat development and maturation phases can significantly explain the quality traits of meat originating from different breeds, muscle types, rearing practices, and post-slaughter processing conditions. Liu and colleagues (2018)^60^ leveraged gene expression data to pinpoint potential biomarkers capable of differentiating between Min (a Chinese breed of pig) and Large White pigs: genes such as ACSM3 (acyl-CoA synthetase medium chain family member 3), HOXC6 (homeobox C6), and ISLR2 (immunoglobulin superfamily containing leucine-rich repeat 2), with noteworthy strong expression for the Min breed in the Biceps femoris muscle relative to the Longissimus dorsi muscle: including CPT1A (carnitine palmitoyltransferase 1A), CPT1B (carnitine palmitoyltransferase 1B), and CRYAB (alpha-crystallin B chain; DJ-1). In a similar vein, differences in lipid accumulation and intramuscular fat (IMF) content in the Longissimus dorsi muscle of Yunling cattle, engaging genes involved in glucose metabolism (PGM1 (phosphoglucomutase 1), GALM (galactose mutarotase), GPI (glucose-6-phosphate isomerase), and LDHA (lactate dehydrogenase A)), and Chinese Simmental cattle, with respect to lipolysis and oxidative metabolism related genes (ALDH9A1 (aldehyde dehydrogenase 9 family member A1), ACSL5 (acyl-CoA synthetase long chain family member 5), ACADM (acyl-CoA dehydrogenase medium chain), ACAT2 (acetyl-CoA acetyltransferase 2), and ACOT2 (acyl-CoA thioesterase 2)), have been identified by Zhang et al. (2018). The tenderness of the longissimus dorsi obtained from Maremmana and Chianina cattle has also been explained in terms of gene expression of various proteins such as TRIM45 (tripartite motif containing 45) related to growth, cellular differentiation, and apoptosis, TRIM32 (tripartite motif containing 32) involved in the regulation of skeletal muscle differentiation.

Additionally, the impact of dietary composition on gene expression and its subsequent influence on meat quality has been investigated by Chen et al. (2019)^61^. In their study on Landrace x Yorkshire pigs, the quantities of mulberry leaves in the feed affected the expression of genes involved in fatty acid metabolism, such as ACOT4 (acyl-coenzyme A thioesterase 4), ECHS1 (enoyl-coa hydratase, short chain 1), HACD1 (3-hydroxyacyl-CoA dehydratase 1), NPR1 (natriuretic peptide receptor 1), ADCY2 (adenylyl cyclase type 2), MGLL (monoglyceride lipase), and IRS1 (insulin receptor substrate 1), as well as those involved in muscle formation and development, including TNNC1 (troponin C1, slow skeletal and cardiac muscles), MYL3 (myosin light chain 3), TCAP (titin-cap), and TNNT1 (troponin T1, slow skeletal type).Consequently, the authors suggested that these could explain observed differences in the longissimus dorsi’s drip loss and shear force.

In the study, PCA and Heat Map analyses were conducted using gene expressions postulated as biomarkers for meat quality in Holstein and Simmental cattle populations raised under conventional and intensive production systems. Significant differences in the expression levels of many of these genes between the groups were identified. Further, these gene expressions were subjected to Pearson correlation analysis, utilizing the results for MDA and CM-H2DCFDA staining as indicators of oxidative stress in the blood. The findings concentrated on 9 genes. With increasing levels of oxidative stress in the blood, the expression of genes involved in muscle development such as CPT1, and those defined as biomarkers for meat tenderness like TRIM32 and PRKAG3, as well as TNNC1 linked to shear force, were downregulated, exhibiting a strong negative correlation. Conversely, expressions of genes associated with oxidative stress biomarkers in meat, such as CRYAB, HSPB1, GPX3, and pro-apoptotic genes BAX and Caspase 3, displayed a significant positive correlation with the levels of oxidative stress in the blood. Taken together, the results indicate that oxidative stress ascertained in post-mortem blood may potentially affect meat quality under postmortem conditions, at least for the biomarker parameters.

Our study findings indicate that cattle subjected to traditional pasture-based rearing, characterized by a natural diet and interaction with a varied array of environmental microbes and other stressors, may exhibit enhanced immunological resilience and climatic adaptability, potentially providing increased resistance to stress prior to slaughter. Moreover, molecular signaling pathways related to oxidative stress, as well as determined meat quality parameters within muscle tissue, suggest that cattle reared in intensive farming systems, which typically have limited human contact and are housed in controlled environments with high-concentrate, additive-rich diets, may be more prone to stress susceptibility during transportation and within holding facilities, a factor that might adversely affect meat quality post-slaughter.

In conclusion, the study results suggest that intensive agricultural practices may worsen the susceptibility of livestock populations to adverse pre-slaughter conditions, with some influence from the breed of cattle. This emphasizes the need for a reassessment of current farming methodologies to ensure the wellbeing of animals in the agricultural sector. The heightened sensitivity observed is likely associated with the pronounced oxidative stress within these groups, potentially impacting apoptotic pathways. The resulting molecular changes driven by such stress have the potential to significantly affect meat quality parameters. This evidence highlights a crucial connection between rearing practices, stress-induced molecular responses, and the overall quality of meat, emphasizing the necessity for further investigation and potential interventions to mitigate the effects of intensive farming on animal welfare and meat quality.

## Materials and methods

### Experimental cohort

For this study, blood and meat samples were collected from the carcasses during and immediately after the slaughter process. The study included a total of 80 bovine animals, with 20 males selected from each of the Holstein (black and white) and Simmental (red and white) breeds that were sourced from farms practicing both conventional and intensive meat production methods, totaling 40 animals from each breed over the course of the trial. Additionally, 5 animals from each breed that were slaughtered in their respective barns during the qourban of sacrifice served as control and adjustment groups in the experiment. The rearing method applied to each animal was identified based on their ear tag numbers. Alongside, retrospective data regarding rearing and transport conditions were collected and documented. Transport distances for the experiment groups were restricted to between 50–100 km, and only animals that remained at the collection site for a minimum of 12–16 h were included in the study.

### Blood and serum collection protocol

During the slaughtering process, blood was drawn from the common carotid artery into aseptic collection bags. The samples were then apportioned into tubes with yellow caps for serum separation and purple caps containing ethylenediaminetetraacetic acid (EDTA) designated for genetic assays. The serum-containing tubes were allowed to stand at room temperature for half an hour, followed by centrifugation at 3,000 revolutions per minute for 10 min. The resulting serum was then aliquoted into sterile Eppendorf tubes and preserved in liquid nitrogen with pertinent label data. The blood samples in EDTA-treated vials were secured in screw-capped aluminum cryotubes and subsequently submerged in liquid nitrogen within a Dewar vessel. These samples were transported to the laboratory in liquid nitrogen for ultra-low temperature storage at -80 °C pending analysis.

### Meat sample procurement

Subsequent to slaughter, samples from the *Musculus longissimus* dorsi were extracted an hour post mortem a timeframe coinciding with the meat packing or transport phase. Utilizing a biopsy punch, three specimens were taken from each carcass. These samples were deposited into sterile cryotubes with screw caps and were immediately frozen in liquid nitrogen. They were stowed at -80 °C until subsequent analyses were conducted.

### Quantification of lipid peroxidation and total oxidative stress biomarkers in serum

To ascertain the extent of lipid peroxidation within serum samples, malondialdehyde (MDA) levels were measured. An aliquot of 200 μl of serum was thoroughly mixed with 2 mL of 0.1% trichloroacetic acid (TCA) (Merck, USA) and centrifuged for 5 min at 10,000 rpm using a centrifuge equipped with a 24 × 1.5/2.0 mL rotor. Subsequently, a volume of 2 mL of 0.5% thiobarbituric acid (TBA) (Merck, USA) , prepared in a 20% TCA solution, was combined with the supernatant and subjected to incubation at 95 °C for 30 min within a thermostatic water bath. To terminate the reaction, sample tubes were placed into an ice bath. The mixture was then centrifuged at 10,000 rpm for an additional 15 min. The absorbance of the clear supernatant was recorded at wavelengths of 532 nm and 600 nm employing a spectrophotometer. The MDA concentration was computed using a molar extinction coefficient of 155 mM^ − 1 cm^ − 1 and expressed as nmol/ml of protein.

The assessment of global oxidative stress within the serum was performed using the CM-H2DCFDA assay kit (Thermo Scientific, USA), a fluorescent indicator of oxidative stress. For this assay, 200 µL of serum was treated with 10 µL of CM-H2DCFDA reagent in a 96-well microplate. Samples were incubated at 37 °C, shielded from light, for 30 min. Subsequently, 100 µL was taken, pipetted onto Tali slides, and loaded into the device. The fluorescent product of the reaction was quantified using an excitation wavelength of 480 nm and an emission wavelength of 530 nm with a TALI image-based cytometer. The spectral data were processed utilizing the accompanying software of the instrument, and oxidative stress levels were quantified in relative fluorescence units (rfu).

### Analysis of gene expression in blood and muscle tissues

Total RNA was extracted from 200 µl of the blood samples collected in EDTA tubes for the evaluation of gene expression profiles in blood tissues. Prior to RNA extraction from muscle tissues, samples harvested from the Musculus longissimus dorsi were homogenized. One biopsy punch from each specimen was transferred into 2 ml sterile Eppendorf tubes, and 500 µl of ultrapure water was added followed by thorough homogenization using a Daihan homogenizer. Subsequently, 200 µl of this tissue homogenate was processed for RNA extraction.

Totally 200 ul of both blood samples and muscle homogenates were processed for RNA extraction by adding 1000 µl of Trizol reagent into separate Eppendorf tubes and following the protocol prescribed for Trizol-based RNA isolation. RNA yield, purity, and integrity were quantified using a NanoDrop spectrophotometer, and samples were normalized with ultrapure water. cDNA was synthesized from the isolated RNA using a High Capacity cDNA Reverse Transcription Kit (Applied Biosystems) as per the manufacturer’s instructions, and the resulting cDNA samples were stored at -20 °C until further analysis.

Gene expression analyses were conducted on these cDNA templates, focusing initially on genes associated with oxidative stress, DNA damage repair, molecular chaperone responses within the heat shock protein family, and the apoptosis pathway in blood tissues. For oxidative stress-related gene expression, assays were designed for cytosolic CuZn-SOD, mitochondrial MnSOD, catalase, APX, GS, and GST. To assess DNA damage repair mechanisms, NEIL, SMUG1, XRCC3, and EXO1 were selected. Chaperone activity was evaluated by quantifying HSP27, HSP60, HSP70, and HSP90 expression. Within the context of apoptosis, the mitochondrial pathway was examined through quantification of tumor suppressor P53, anti-apoptotic factors BCL-2, BCL-XL, XIAP, and pro-apoptotic elements Puma, NOXA, BAX, APAF1, Cyt-C, and Caspase 3. Detailed information on the genes, primer sequences, and PCR conditions for the qRT-PCR assay is provided in Supplementary Table S1.

In relation to meat quality parameters, gene expressions pertinent to muscle phenotype and postmortem changes were assayed, including AMD1, CPT1A, CPT1B, CRYAB, GPX3, HSPB1, IRS1, PPARA, PPARGC1A, PYGM, RASGRP3, UCP3, and ZIC1 associated with muscle/slaughter discrepancies (Liu et al. 2018). Additionally, genes influencing intramuscular fat (IMF) and fatty acid composition ALDH9A1, ACSL5, ACADM, ACAT2, ACOT2; and those implicated in metabolic pathways PGM1, GALM, GPI, LDHA (37) were analyzed. Morphological traits related to meat tenderness, such as TRIM45, TRIM32, and PRKAG3, as well as those contributing to shear force TNNC1, MYL3, TCAP, TNNT1^61,62^, were also evaluated (Table S1).

Gene expression levels were determined with Quant Studio 5 Real-Time PCR System (qPCR). The synchronized cDNA templates were aliquoted into 384-well PCR plates, and specific forward and reverse primers for each gene were added. Power SYBR Green PCR Master Mix was utilized for qRT-PCR amplification, with the thermocycling conditions set to one cycle at 50 °C for 2 min and 95 °C for 10 min, followed by 40 cycles of denaturation at 95 °C for 15 s, and annealing and extension at 60 °C for 1 min. The Ct (cycle threshold) values generated during amplification were utilized to quantify gene expression, employing the 2^−∆∆Ct method for calculation. Glyceraldehyde 3-phosphate dehydrogenase (GAPDH) and β-actin were used as reference genes for normalization and correction of expression data.

### Western blot analysis

For the isolation of proteins from meat tissues, the RIPA Lysis Buffer System (Santa Cruz Biotechnology) kit protocol was followed. Protein quantities were standardized using the Qubit® Fluorometer (Invitrogen), and they were subsequently loaded onto NuPAGE™ (Life Technologies) gels. In this study, WesternBreeze chemiluminescent kits were utilized, with the blotting and membrane transfer processes conducted using the iBlot (Life Technologies) system with ready-made membranes and kits. Following blotting, proteins were treated with primary antibodies including Bcl-2 Antibody (C-2), caspase-3 Antibody (31A1067), Bax (B-9): sc-7480 (Santa Cruz, USA), and β-Actin Monoclonal Antibody (ACTN05 (C4)) (ThermoFisher Scientific). Afterward, the antibodies were labeled with appropriate secondary antibodies and visualized through chemiluminescence imaging with ChemiDoc. Protein quantities were determined using the device’s existing software.

### Statistical analysis

In this study, several statistical methods were employed to analyze the data. Differences in mean MDA levels, oxidative stress staining results, and gene expression values were assessed using one-way ANOVA. When the effects of rearing (farming systems) conditions on any of the variables were significant (p ≤ 0.05) after ANOVA, their means were separated using Tukey’s HSD test to identify significant differences. Pearson correlation analysis was conducted to examine relationships between measured parameters, with a significance level set at p ≤ 0.05 for all statistical tests. All analyses were performed using SPSS version 25^63^.

To investigate the relationship between rearing conditions and meat quality parameters, as well as blood gene expressions, principal component analysis (PCA) was first conducted to reduce dimensionality and visualize the data structure. First of all, Kaiser Meyer Olkin (KMO) sampling adequacy value was found to be 0.581 and it was seen that the sample size was sufficient^64^. As a result of Barlett’s test, χ2(378) = 2933,388, p < 0.05 and this finding showed that the correlations between the items were large enough. It was determined that a total of 28 items consisted of two sub-dimensional constructs and explained approximately fifty percent (50%) of the total variance. Hierarchical cluster analysis was then performed using Euclidean distance and Ward’s method to differentiate between groups based on the identified parameters, using MetaboAnalyst 4.0 software.

## Supplementary Information

Supplementary Table S1.

Supplementary Figure.

## Data Availability

The datasets used and analysed during the current study available from the corresponding author on reasonable request.

## References

[1] Galka, A. Using a cleaner production preventive strategy for the reduction of the negative environmental impacts of agricultural production using cattle husbandry as a case study. *J. Clean. Prod.* **12**(5), 513–516 (2004).

[2] Erb, K. H. et al. Exploring the biophysical option space for feeding the world without deforestation. *Nat. Commun.* **7**(1), 1–9 (2016). 10.1038/ncomms11382 PMC4838894 27092437

[3] Ruviaro, C. F. et al. Economic and environmental feasibility of beef production in different feed management systems in the Pampa biome, southern Brazil. *Ecol. Ind.* **60**, 930–939 (2016).

[4] Ahlgren, S. et al. Climate and biodiversity impact of beef and lamb production–A case study in Sweden. *Agric. Syst.* **219**, 104047 (2024).

[5] Bragaglio, A. et al. Environmental impacts of Italian beef production: A comparison between different systems. *J. Clean. Prod.* **172**, 4033–4043 (2018).

[6] Gill, M., Smith, P. & Wilkinson, J. M. Mitigating climate change: the role of domestic livestock. *Animal* **4**(3), 323–333 (2010). 22443938 10.1017/S1751731109004662

[7] Webb, E. C. & Erasmus, L. J. The effect of production system and management practices on the quality of meat products from ruminant livestock. *South Afr. J Anim. Sci.* **43**(3), 413–423 (2013).

[8] Sumberg, J. & Giller, K. E. What is ‘conventional’ agriculture?. *Glob. Food Secur.* **32**, 100617 (2022).

[9] McAllister, T. A. et al. Nutrition, feeding and management of beef cattle in intensive and extensive production systems Animal agriculture (75–98). (Academic Press (2020).

[10] Mondière, A. et al. Trade-offs between higher productivity and lower environmental impacts for biodiversity-friendly and conventional cattle-oriented systems. *Agric. Syst.* **213**, 103798 (2024).

[11] Aquilani, C., Confessore, A., Bozzi, R., Sirtori, F. & Pugliese, C. Precision livestock farming technologies in pasture-based livestock systems. *Animal* **16**(1), 100429 (2022). 34953277 10.1016/j.animal.2021.100429

[12] Hermansen, Z. E. & Zervas, G. Round Table discussion of the organic animal production session. *Livest. Sci.* **90**, 63–65 (2004).

[13] Sundrum, A. Organic livestock farming: a critical review. *Livest. Prod. Sci.* **67**(3), 207–215 (2001).

[14] Blanco-Penedo, I. et al. Evaluation of food safety and quality in organic beef cattle in NW Spain; a comparison with intensive and conventional systems. *Agron. Res* **7**(2), 585–591 (2009).

[15] Bittante, G. et al. Veal and beef meat quality of crossbred calves from dairy herds using sexed semen and semen from double-muscled sires. *Ital. J. Anim. Sci.* **22**(1), 169–180. 10.1080/1828051X.2023.2171919 (2023).

[16] Domingo, G. et al. Effect of crossbreeding with Limousine, Rubia Gallega and Belgium Blue on meat quality and fatty acid profile of Holstein calves. *Anim. Sci. J.* **86**(11), 913–921 (2015). 25706373 10.1111/asj.12373

[17] Gagaoua, M., Monteils, V., Couvreur, S. & Picard, B. Identification of biomarkers associated with the rearing practices, carcass characteristics, and beef quality: An integrative approach. *J. Agric. Food Chem.* **65**(37), 8264–8278 (2017). 28844145 10.1021/acs.jafc.7b03239

[18] Franco, D., Carballo, J., Bermñudez, R. & Lorenzo, J. M. Effect of genotype and slaughter age on carcass traits and meat quality of the Celta pig breed in extensive system. *Anna. Anim. Sci.* **16**(1), 259–273 (2016).

[19] Liu, W. et al. Effects of dietary Allium mongolicum Regel powder supplementation on the growth performance, meat quality, antioxidant capacity and muscle fibre characteristics of fattening Angus calves under heat stress conditions. *Food Chem.* **453**, 139539 (2024). 38788638 10.1016/j.foodchem.2024.139539

[20] Qin, X. et al. Effects of dietary sea buckthorn pomace supplementation on skeletal muscle mass and meat quality in lambs. *Meat sci.* **166**, 108141 (2020). 32302933 10.1016/j.meatsci.2020.108141

[21] Whyte, H. et al. "The potential of the mineral composition to discriminate between beef from different cattle diets and between individual muscles. *Food Control* **163**, 110539 (2024).

[22] Gagaoua, M., Monteils, V. & Picard, B. Decision tree, a learning tool for the prediction of beef tenderness using rearing factors and carcass characteristics. *J. Sci. Food Agric.* **99**(3), 1275–1283 (2019). 30073653 10.1002/jsfa.9301

[23] Maggiolino, A. et al. Carcass and meat quality characteristics from Iberian wild red deer (Cervus elaphus) hunted at different ages. *J. Sci. Food Agric.* **99**(4), 1938–1945 (2019). 30270485 10.1002/jsfa.9391

[24] Acevedo-Giraldo, J. D., Sánchez, J. A. & Romero, M. H. Effects of feed withdrawal times prior to slaughter on some animal welfare indicators and meat quality traits in commercial pigs. *Meat sci.* **167**, 107993 (2020). 32388087 10.1016/j.meatsci.2019.107993

[25] Bogdanowicz, J., Cierach, M. & Żmijewski, T. Effects of aging treatment and freezing/thawing methods on the quality attributes of beef from Limousin× Holstein-Friesian and Hereford× Holstein-Friesian crossbreeds. *Meat sci.* **137**, 71–76 (2018). 29154221 10.1016/j.meatsci.2017.10.015

[26] Contini, C. et al. Effect of an active packaging with citrus extract on lipid oxidation and sensory quality of cooked turkey meat. *Meat Sci.* **96**(3), 1171–1176 (2014). 24334037 10.1016/j.meatsci.2013.11.007

[27] Palmieri, B. & Sblendorio, V. Oxidative stress tests: overview on reliability and use. *Eur. Rev. Med. Pharmacol. Sci.* **11**(6), 383–399 (2007). 18306907

[28] Kreutzmann, M. et al. Differential modulation of markers of oxidative stress and DNA damage in arterial hypertension. *Antioxidants*10.3390/antiox12111965 (2023). 38001818 10.3390/antiox12111965 PMC10669810

[29] McCord, J. M. The evolution of free radicals and oxidative stress. *Am. J. Med.* **108**(8), 652–659 (2000). 10856414 10.1016/s0002-9343(00)00412-5

[30] Rock, C. L., Jacob, R. A. & Bowen, P. E. Update on the biological characteristics of the antioxidant micronutrients: vitamin C, vitamin E, and the carotenoids. *J. Am. Diet. Assoc.* **96**(7), 693–702 (1996). 8675913 10.1016/S0002-8223(96)00190-3

[31] Cataldi, A. Cell responses to oxidative stressors. *Curr. Pharm. Design* **16**(12), 1387–1395 (2010). 10.2174/138161210791033969 20166986

[32] Fayemi, P. O. & Muchenje, V. Meat in African context: From history to science. *Afr. J. Biotechnol.* **11**(6), 1298–1306 (2012).

[33] Minka, N. S. & Ayo, J. O. Physiological responses of food animals to road transportation stress. *Afr. J. Biotechnol.* **8**, 25 (2009).

[34] Sullivan, P. A. et al. Preslaughter factors affecting mobility, blood parameters, bruising, and muscle pH of finished beef cattle in the United States. *Trans. Anim. Sci.*10.1093/tas/txae035 (2024). 10.1093/tas/txae035 PMC10983080 38562213

[35] Warriss, P. D. Meat science. CABI publishing (2010).

[36] Chulayo, A. Y., Tada, O. & Muchenje, V. Research on pre-slaughter stress and meat quality: A review of challenges faced under practical conditions. *Appl. Anim. Husb. Rural Dev.* **5**, 1–6 (2012).

[37] Mathew, O. A., Foluke, A., Olufemi, M. A., Opeyemi, A. & Micheal, A. Comparative effect of vitamin complex and orange extract on physiological and blood parameters of transported pullets in humid tropics. *Online J. Anim. Feed Res.* **13**(2), 97–104. 10.51227/ojafr.2023.15 (2023).

[38] Fayemi, P. O. & Muchenje, V. Maternal slaughter at abattoirs: history, causes, cases and the meat industry. *SpringerPlus* **2**(1), 1–7 (2013). 23577300 10.1186/2193-1801-2-125 PMC3618883

[39] Chulayo, A. Y. & Muchenje, V. Effect of pre-slaughter conditions on physico-chemical characteristics of mutton from three sheep breeds slaughtered at a smallholder rural abattoir. *South Afr. J. Anim. Sci.* **43**(5), 64–68 (2013). 10.5713/ajas.2013.13141 PMC4092882 25049767

[40] Muchenje, V., Dzama, K., Chimonyo, M., Strydom, P. E. & Raats, J. G. Relationship between pre-slaughter stress responsiveness and beef quality in three cattle breeds. *Meat Sci.* **81**(4), 653–657 (2009). 20416575 10.1016/j.meatsci.2008.11.004

[41] Ferguson, D. M. & Warner, R. D. Have we underestimated the impact of pre-slaughter stress on meat quality in ruminants?. *Meat Sci.* **80**(1), 12–19 (2008). 22063165 10.1016/j.meatsci.2008.05.004

[42] Piccione, G. et al. Oxidative stress associated with road transportation in ewes. *Small Rumin. Res.* **112**(1–3), 235–238 (2013).

[43] Costantini, D. & Bonadonna, F. Patterns of variation of serum oxidative stress markers in two seabird species. *Polar Res.* **29**(1), 30–35 (2010).

[44] Mapiye, C. et al. The labile lipid fraction of meat: From perceived disease and waste to health and opportunity. *Meat sci.* **92**(3), 210–220 (2012). 22546816 10.1016/j.meatsci.2012.03.016

[45] Sazili, A. Q. et al. Quality assessment of longissimus and semitendinosus muscles from beef cattle subjected to non-penetrative and penetrative percussive stunning methods. *Asian-Australas. J. Animal Sci.* **26**(5), 723–731 (2013). 10.5713/ajas.2012.12563 PMC4093328 25049845

[46] Doganlar, O. & Doganlar, B. Z. Responses of antioxidant enzymes and heat shock proteins in Drosophila to treatment with a pesticide mixture. *Arch. Biol. Sci.* **67**(3), 869–876 (2015).

[47] Doganlar, O., Doganlar, Z. B., Muranlı, F. D. G. & Guner, U. Genotoxic effect and carcinogenic potential of a mixture of as and cd in zebrafish at permissible maximum contamination levels for drinking water. *Water Air Soil Pollut.* **227**(3), 1–16 (2016).

[48] Munekata, P. E., Pateiro, M., López-Pedrouso, M., Gagaoua, M. & Lorenzo, J. M. Foodomics in meat quality. *Current Opin. Food Sci.* **38**, 79–85 (2021).

[49] Lamri, M. et al. Towards the discovery of goat meat quality biomarkers using label-free proteomics. *J. Proteom.* **278**, 104868 (2023). 10.1016/j.jprot.2023.104868 36871648

[50] Güçlü, H. et al. Effects of cisplatin-5-fluorouracil combination therapy on oxidative stress, DNA damage, mitochondrial apoptosis, and death receptor signalling in retinal pigment epithelium cells. *Cutan. Ocular Toxicol.* **37**(3), 291–304 (2018). 10.1080/15569527.2018.1456548 29606027

[51] Doğanlar, Z. B. et al. The role of melatonin in oxidative stress, DNA damage, apoptosis and angiogenesis in fetal eye under preeclampsia and melatonin deficiency stress. *Curr. Eye Res.* **44**(10), 1157–1169 (2019). 31090463 10.1080/02713683.2019.1619778

[52] Lykkesfeldt, J. & Svendsen, O. Oxidants and antioxidants in disease: oxidative stress in farm animals. *Vet. J.* **173**(3), 502–511 (2007). 16914330 10.1016/j.tvjl.2006.06.005

[53] Wiseman, H. & Halliwell, B. Damage to DNA by reactive oxygen and nitrogen species: role in inflammatory disease and progression to cancer. *Biochem. J.* **313**(1), 17–29 (1996). 8546679 10.1042/bj3130017 PMC1216878

[54] Huang, F. et al. Changes in apoptotic factors and caspase activation pathways during the postmortem aging of beef muscle. *Food Chem.* **190**, 110–114 (2016). 26212948 10.1016/j.foodchem.2015.05.056

[55] Kurashova, N. A., Madaeva, I. M. & Kolesnikova, L. I. Expression of HSP70 heat-shock proteins under oxidative stress. *Adv. Gerontol.* **10**, 20–25 (2020). 31800176

[56] Vardar, S. A. et al. Different responses of apoptotic, inflammatory and heat shock protein gene expression to a single bout of high-intensity interval exercise between physically active and inactive men. *Appl. Physiol. Nutr. Metab.* **46**(7), 743–752 (2021). 33439763 10.1139/apnm-2020-0783

[57] Zhang, H. M. et al. Longissimus dorsi muscle transcriptomic analysis of Yunling and Chinese simmental cattle differing in intramuscular fat content and fatty acid composition. *Genome* **61**(8), 549–558 (2018). 29883552 10.1139/gen-2017-0164

[58] Archile-Contreras, A. C. & Purslow, P. P. Oxidative stress may affect meat quality by interfering with collagen turnover by muscle fibroblasts. *Food Res. Int.* **44**(2), 582–588 (2011).

[59] Falowo, A. B., Fayemi, P. O. & Muchenje, V. Natural antioxidants against lipid–protein oxidative deterioration in meat and meat products: A review. *Food Res. Int.* **64**, 171–181 (2014). 30011637 10.1016/j.foodres.2014.06.022

[60] Liu, Y. et al. Transcriptomics analysis on excellent meat quality traits of skeletal muscles of the Chinese indigenous min pig compared with the large white breed. *Int. J. Mol. Sci.* **19**(1), 21 (2018). 10.3390/ijms19010021 PMC5795972 29271915

[61] Chen, G., Su, Y., Cai, Y., He, L. & Yang, G. Comparative transcriptomic analysis reveals beneficial effect of dietary mulberry leaves on the muscle quality of finishing pigs. *Vet. Med. Sci.* **5**(4), 526–535 (2019). 31486291 10.1002/vms3.187 PMC6868455

[62] Wang, L., Shi, H., Huang, J.-L., Xu, S. & Liu, P. P. Linggui Zhugan decoction inhibits ventricular remodeling after acute myocardial infarction in mice by suppressing TGF-β_1_/Smad signaling pathway. *Chin. J. Integr. Med.* **26**(5), 345–352 (2020). 30623345 10.1007/s11655-018-3024-0

[63] SPSS SPSS for Windows, Version 25.0, SPSS Inc. Chicago (2024).

[64] Field, A. *Discovering Statistics Using IBM SPSS Statistics*, 5th ed.; SAGE Publications Ltd.: London, UK; Philadelphia, PA, USA, (2009).

