# Supplementary material for: Effects of intensive and conventional farming on oxidative stress and meat quality biomarkers in holstein and simmental cattle
Source: Sci Rep. 2024 Oct 31;14:26197. doi: 10.1038/s41598-024-78087-x (PMC11526110; doi:10.1038/s41598-024-78087-x)
Supplement: Supplementary file 1 — Supplementary Table S1. [file 41598_2024_78087_MOESM1_ESM.pdf]

Table S1. Genes, primer sequences, and PCR conditions for the qRT-PCR assay.

Gene ID	Gene name			Sequences (5'-3')	
**Antioxidant defence signals**					
**SOD1**	CuZn-Superoxide dismutase	F	5’	CACGATGGTGGTCCATGAA	3’
		R	5’	TTCCAGCGTTGCCAGTCTT	3’
**SOD2**	Mn-Superoxide dismutase	F	5’	ACGTGAACAACCTCAACGT	3’
		R	5’	AGTCACGTTTGATGGCTTC	3’
**CAT**	Catalase	F	5’	GTTCGCTTCTCCACTGTT	3’
		R	5’	GGCCATAGTCAGGATCTT	3’
**GST**	Glutation-S-transferase	F	5’	TTCCCTCTGCTAAAGGCCC	3’
		R	5’	CTTCCTCTGGCTGCCAGG	3’
**DNA repair signals**					
**EXO1**	Exonuclease 1	F	5’	GCAACTTCTTCGTGAGGGA	3’
		R	5’	AGGAAGGTATTGTTGGCCC	3’
**XRCC3**	X-Ray Repair Cross-Complementing Gene 3	F	5’	GGTCGAGTGACAGTCCAA	3’
		R	5’	TGCAACGGCTGAGGGTCTT	3’
**SMUG1**	Single-strand-selective monofunctional uracil-DNA glycosylase 1	F	5’	AATGATGAGCTGAGGCAA	3’
		R	5’	GCGTATTCCACAGGGTTGT	3’
**MLH1**	mutL homolog 1	F	5’	GAAATATCATTGCTCACGC	3’
		R	5’	CAGATGGAGTGGGAGAT	3’
**Heat Shock Protein families (HSPs)**					
**HSP27**	Heat shock protein family B (small) member 1	F	5’	GCTGAATAAAACCCGACA	3’
		R	5’	CAAGCAAGATCGAACCCT	3’
**HSP60**	Heat shock protein family D (Hsp60) member 1	F	5’	CTCATCTCACTCGGGCTTA	3’
		R	5’	GGCTACAGCATCGGCTAA	3’
**HSP70**	Heat shock protein 70	F	5’	GACGACGGCATCTTCGAG	3’
		R	5’	GTTCTGGCTGATGTCCTTC	3’
**HSP90**	Heat shock protein 90 alpha family class A member 1	F	5’	TGGTGGCTGAGAAAGTGA	3’
		R	5’	TGTTCCACGTCCCATTGGT	3’
**Apoptosis signaling**					
**BCL-2**	B-cell CLL/lymphoma 2	F	5’	GAGTCGGATCGCAACTTGG	3’
		R	5’	CTCTCGGCTGCTGCATTGT	3’
**BAX**	BCL2-associated X protein	F	5’	TCTGACGGCAACTTCAACT	3’
		R	5’	GGTGTCCCAAAGTAGGAG	3’
**Cyt-C**	Cytochrome c	F	5’	TGCTGGTGATGTTGAGAAG	3’
		R	5’	GTGTCCTCGTTCCAGGTGA	3’
**Caspase 3**	Caspase 3	F	5’	AACCTCCGTGGATTCAAAA	3’
		R	5’	TTCAGGRTAATCCATTTTG	3’
**Caspase 8**	Caspase 8	F	5’	TGTCACAATCGCTTCCAGA	3’
		R	5’	GAAGTTCAGGCACCTGCTT	3’
**Meat quality potential biomarkers**					
**AMD1**	Adenosylmethionine decarboxylase 1	F	5’	TCCACAAGTCAAGTCCTCT	3’
		R	5’	CCATGGAGAGGAACGAAT	3’
**CPT1A**	Carnitine palitoyltransferase 1A	F	5’	ACAAGCCATAGTCTTAACG	3’
		R	5’	GCCAGTCCAGGATAACAA	3’
**CPT1B**	Carnitine palmitoyltransferase 1B	F	5’	ACTGTCTGGGCAAACCAA	3’
		R	5’	CTTCTTGATGAGGCCTTTG	3’

**IRS1**	Insulin receptor substrate 1	F<br>R	5’<br>5’	TGCCTGACCAGCAAGACC<br>ATCCACCTGCATCCAAAAC	3’<br>3’
**CRYAB**	alpha-crystallin B chain; DJ-1	F<br>R	5’<br>5’	GACCCTCTCACCATTACTT<br>CAGCAGGCTTCTCTTCACG	3’<br>3’
**UPC3**	Uncoupling protein 3	F<br>R	5’<br>5’	GACGTGGTGAAGGTTCGAT<br>CGAGTTCATGTACCGGGTC	3’<br>3’
**HSPB1**	Heat shock protein family B (small) member 1	F<br>R	5’<br>5’	CCTGTCACTTTCGAGGCG<br>AGGTGGGGATGGCTGGT	3’<br>3’
**GPX3**	Glutathione peroxidase 3	F<br>R	5’<br>5’	GCTTCCCCTGCAACCAATT<br>GGACATACCTGAGAGTGG	3’<br>3’
**PGM1**	Phosphoglucomutase 1	F<br>R	5’<br>5’	GATCCTGTGGACGGAAGC<br>ATGTACAGTCGGATGGTGG	3’<br>3’
**GALM**	Galactomutarotase	F<br>R	5’<br>5’	AACCGAATTGCCAAAGGA<br>TCACCATCTGGACTGACCC	3’<br>3’
**GPI**	Glucose-6-phosphate isomerase	F<br>R	5’<br>5’	CGGAGTGGCGAATGGAAA<br>GAGGCAATGATGAACAGG	3’<br>3’
**LDHa**	Lactate dehydrogenase A	F<br>R	5’<br>5’	ATCTTGACCTATGTCGCTT<br>TCTTCAGGGAGACACCAGC	3’<br>3’
**ALDH9A1**	Aldehyde dehydrogenase 9 family member A1	F<br>R	5’<br>5’	ATCGGAGCTGTAGCCA<br>TGGCCTATGCCAGAGC	3’<br>3’
**ACOT2**	Acyl-CoA thioesterase 2	F<br>R	5’<br>5’	CTACCTGCTTAATCACCCT<br>GCGGCATAATCTCACCTT	3’<br>3’
**ACSL5**	Acyl-CoA synthetase long chain family member 5	F<br>R	5’<br>5’	CCCTACAGATGGCTGTCCT<br>GCTCCCAAGGTGTCATACA	3’<br>3’
**ACADM**	Acyl-CoA dehydrogenase medium chain	F<br>R	5’<br>5’	GCGAGTACCCTGTCCCATT<br>CCTCAGTCATTCTCCCCAA	3’<br>3’
**ACAT2**	Acetyl-CoA acetyltransferase 2	F<br>R	5’<br>5’	ATCACCAAGGAGCGAATC<br>CCTCTTCTGCTTGTCCCAA	3’<br>3’
**TRIM32**	Tripartite motif containing 32	F<br>R	5’<br>5’	CAGGCCAGGTATAAAGCA<br>CTCGGCAATGTTAAGCAGG	3’<br>3’
**PRKAG3**	Protein kinase AMP-activated non-catalytic subunit gamma 3	F<br>R	5’<br>5’	CTCCGACTCCAACACAGAC<br>TTCTGCAGCTCATCATCCC	3’<br>3’
**TNNC1**	Troponin C1	F<br>R	5’<br>5’	GATGACATCTACAAGGCTG<br>GCACGAAGATGTCAAAGG	3’<br>3’
**TNNT1**	Troponin T1	F<br>R	5’<br>5’	CATCCGCGGTTTAGGAATC<br>CCCCCTCTGGGATCTTCGG	3’<br>3’
**MYL3**	Myosin light chain 3	F<br>R	5’<br>5’	AGGACTTTGTGGAAGGGCT<br>TCTTGCCCAGCCATCAACT	3’<br>3’
**TCAP**	Ttitin-cap	F<br>R	5’<br>5’	CGTGAGACCTACCACCAAC<br>CCACTTTGGCAGGGGTGAA	3’<br>3’
**Endogen control**					
**GAPDH**	Gglyceraldehyde-3-phosphate dehydrogenase	F<br>R	5’<br>5’	CTACTCGGGCCTCTTCTGT<br>GATTCTCCCGATCAGTCAG	3’<br>3’
**β-actin**	The beta actin	F<br>R	5’<br>5’	CCACGAAACTACCTTCAAC<br>TGATCTCCTTCTGCATCCT	3’<br>3’
**PCR conditions**					
**1 cycle of 2 min at 50°C and 10 min at 95°C followed by 40 cycles of denaturation at 95°C for 15 s, annealing and extension at 60°C for 1 min**					
