# Supplementary figures and images for: Effects of intensive and conventional farming on oxidative stress and meat quality biomarkers in holstein and simmental cattle
Source: Sci Rep. 2024 Oct 31;14:26197. doi: 10.1038/s41598-024-78087-x (PMC11526110; doi:10.1038/s41598-024-78087-x)

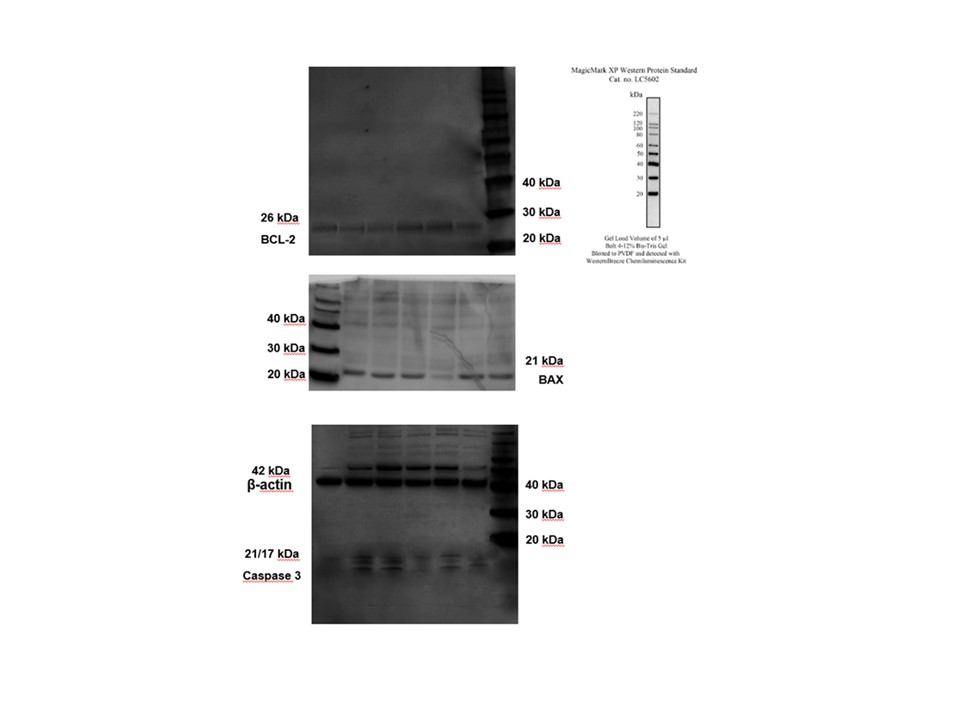

Supplement: Supplementary file 2 — Supplementary Figure. [file 41598_2024_78087_MOESM2_ESM.jpg]
